# Selection of Suitable Reference Genes for qPCR Normalization under Abiotic Stresses in *Oenanthe javanica* (BI.) DC

**DOI:** 10.1371/journal.pone.0092262

**Published:** 2014-03-20

**Authors:** Qian Jiang, Feng Wang, Meng-Yao Li, Jing Ma, Guo-Fei Tan, Ai-Sheng Xiong

**Affiliations:** State Key Laboratory of Crop Genetics and Germplasm Enhancement, Key Laboratory of Biology and Germplasm Enhancement of Horticultural Crops in East China of Ministry of Agriculture, College of Horticulture, Nanjing Agricultural University, Nanjing, People’s Republic China; Florida International University, United States of America

## Abstract

Accurate normalization of gene expression data is an absolute prerequisite to obtain reliable results in qPCR analysis. *Oenanthe javanica,* an aquatic perennial herb, belongs to the *Oenanthe* genus in *Apiaceae* family, with known medicinal properties. In the current study, *O. javanica* was subjected to hormone stimuli (gibberellin, salicylic acid, methyl jasmonate, and abscisic acid) and abiotic stresses (heat, cold, salt, and drought), and the expression of nine candidate reference genes (*eIF-4α, ACT7, TIP41, GAPDH, SAND, EF-1α, PP2A, TBP*, and *TUB*) was evaluated. Stability of the genes was assessed using geNorm, NormFinder and BestKeeper. All the genes presented distinct expression profiles under the experimental conditions analyzed. Under abiotic stress conditions, *ACT7* and *PP2A* genes displayed the maximum stability; *PP2A* and *SAND* were the most stable genes under hormone stimuli. Even though *PP2A* gene was most stable across all the samples, individual analysis revealed changes in expression profile. To further validate the suitability of the reference genes identified in this study, the expression level of *M6PR* gene under salt treatment was studied. Based on our data, we propose that it is essential to normalize the target gene expression with specific reference genes under different experimental conditions for most accurate results. To our knowledge, this is the first systematic analysis for reference genes under abiotic stress and hormone stimuli conditions in *O. javanica*. This will be beneficial for future studies on *O. javanica* and other plants in *Apiaceae* family at molecular level.

## Introduction

Due to its specificity, accuracy, efficiency and reproducibility, Quantitative real-time PCR (qPCR) has become the most prevalent method for quantifying transcript expression levels and confirming gene expression patterns in different cell types under different conditions, including abiotic and biotic stresses [1], [2]. Normalization of the data is a crucial prerequisite for accurate interpretation of the results, especially when minor variations in gene expression are associated with great deal of biological significance [3]. Stability of the expressed gene is a key factor for an appropriate normalization standard. To avoid bias from RNA stability, quantity, purity, and enzymatic efficiency in cDNA synthesis and PCR amplification, the data is normalized to one or more of the reference genes [4], [5]. Choosing the appropriate reference gene that express stably in both the control and experimental conditions is one of the challenges in gene expression analysis [1], [6], [7].

Some of the widely used qPCR reference genes in plants and animals include: *Actin*, 18S ribosomal RNA (*18S rRNA*), Glyceraldehyde-3-phosphate dehydrogenase (*GAPDH*), Elongation factor-1α (*EF-1α*), Polyubiquitin (*UBQ*), and Tubulin (*TUB*) [8]–[10]. These genes are considered as housekeeping genes with roles in basic cellular processes, and referred as traditional reference genes. In addition, some new reference genes were identified and found to express stably. These reference genes include: genes encoding F-box/kelch-repeat protein (*F-box*), SAND family protein (*SAND*), protein phosphatase 2A (*PP2A*), phosphoenolpyruvate carboxylase-related kinase 1 (*PEPKR1*), and Tap42-interacting protein of 41 kDa (*TIP41*) [11]–[13].

Several environmental and growth factors impact the expression of genes involved in biological processes related to the development of plants and affect their biomass. The stresses include light [14], temperature [15], salt [16], drought [17], [18], oxidative stress [19], hormones [20], and infections from pathogens [21] are some of the stress factors that affect the gene expression. As reference genes play significant roles in target gene search, several studies have focused toward determining optimal reference gene candidates for qPCR, both in model and non-model plants [22], [23].

*Oenanthe javanica* (BI.) DC. is an aquatic perennial herb originating from East Asia, and belongs to *Oenanthe* genus in *Apiaceae* family (Fig. S1). Nowadays, the *O. javanica* is a popular vegetable. The *O. javanica* with abundant vitamins and minerals is not only consumed as a vegetable, but has also been used as a medicinal agent. In traditional Chinese medicine, *O. javanica* is recommended as a treatment for jaundice, hypertension, fever, abdominal pain, leucorrhea, mumps, and urinary infections [24]. Several studies have reported pharmacological benefits of *O. javanica*. Hyperoside, persicarin, and isorhamnetin are the three major substances with pharmacological activities in *O. javanica*. These substances are shown to possess hepatoprotective [25], antithrombotic [26], antiarrhythmic [27], anti-diabetic [28], anti-hepatitis B virus [29], neuroprotective [30], and anticancer [31], [32] activities. However, there is no systematic analysis approaching reference genes selection in *O. javanica*, which can pose a challenge for further research in *O. javanica*.

In this study, nine candidate reference genes (*eIF-4α*, *ACT7*, *TIP41*, *GAPDH*, *SAND*, *EF-1α*, *PP2A*, *TBP*, and *TUB*) were selected based on the previous studies that suggested stable expression [8]–[12], [33]. Detail information of these reference genes is listed in Table 1. Expression data of the genes were determined by qPCR in *O. javanica* plants that were subjected to different hormone stimuli (gibberellin, salicylic acid, abscisic acid, and methyl jasmonate) and abiotic stresses (heat, cold, salt, and drought). Three different algorithms (geNorm, NormFinder, and BestKeeper) were used to evaluate the stability of the reference genes. Furthermore, to validate the selection of candidate reference genes in *O. javanica*, the expression level of Mannose-6-phosphate reductase (*M6PR*) gene under salt treatment was assessed using different reference genes. The current work provides basis to select the candidate reference genes, and adds new information to future research on *O. javanica* and other plants in *Apiaceae*.

**Table 1:** Descriptions of candidate reference genes in *O. javanica.*

Gene symbol	Gene name	Arabidopsis homolog locus	Primer sequence (5′–3′) forward/reverse	Amplicon length (bp)	E (%)	Tm(°C)
*eIF-4α*	Eukaryotic translation initiation factor 4α-1 gene	AT3G13920	CCGCTCTGGTTCTTCTCGTGTG/TGGAGGTAGTTCTCTGGCTGAGTC	120	103.0	82.5
*ACT7*	Actin7 gene	AT5G09810	AGAAGCACCACTGAATCCTAAGGC/GCATACACCATCACCAGAGTCCAA	163	100.3	83
*TIP41*	Tap42-interacting protein of 41 kDa gene	AT4G34270	GGCTTAGAGTTGATGGCGTGCTTA/GTGGCTTCTCTCCAGCAACATTCT	112	102.5	81.5
*GAPDH*	Glyceraldehyde-3-phosphate dehydrogenase gene	AT1G42970	CCTTGGGCTGAGATGGGAATCG/TGACCTTCTTTGCTCCTGCTTGG	103	103.2	83
*SAND*	SAND family protein gene	AT2G28390	TCTTCTCGCCCTGGATCTTTGTCA/AGGACCACCTAACAGTGCCTTACA	93	95.0	83
*EF-1α*	Elongation factor -1αgene	AT1G07940	AGCCTCTTCGTCTGCCACTTCA/GCTTCCAGGAGAGCCTCGTGAT	175	94.1	84.5
*PP2A*	Protein phosphatase 2A gene	AT4G15415	TGGCTGACACAGTAATTCGAGGTT/CTGACGGAACAGACGGACCAT	149	94.4	82.5
*TBP*	TATA-box binding ptotein gene	AT1G55520	TGGCGGAACAAGTCTTGGAAGG/TGATTCGCATAATAACGGCAGCAA	192	91.6	83
*TUB*	TUB beta-7 gene	AT2G29550	GCCACCCTTTCTGTTCATCAGTTG/AGGCAGCAGGTAACTCCACTCA	167	98.4	82.5

## Materials and Methods

### Plant Materials and Treatments

Plants were grown in a soil/vermiculite mixture (3∶1) in a controlled-environment growth chamber programmed for 16 h and 8 h at 25°C and 16°C for day and night conditions with 3 000 lux of light intensity. Two-month-old seedlings were irrigated with ddH_2_O, 200 mM NaCl, and 20% PEG 6000 for 2 h in control sample, and salt and drought treated samples. Leaves were sprayed with 500 ppm gibberellins (GA treatment), 200 ppm salicylic acid (SA treatment), 200 ppm methyl jasmonate (MeJA treatment), or 25 ppm abscisic acid (ABA treatment) for 2 h [1], [34], [35]. Cold and heat treatments were performed by placing the pots containing two-month-old seedlings in chambers at 4°C (cold treatment) or 40°C (heat treatment) for 2 h under constant light intensity. Three biological experimental replicates were performed in different pots on different dates for each treatment. Treated leaf samples were harvested for testing, with untreated leaf samples as controls. The materials were collected, quickly immersed in liquid nitrogen and stored at −80°C until RNA extraction.

### RNA Extraction and cDNA Synthesis

Total RNA was extracted using RNAsimply total RNA Kit (Tiangen, Beijing, China) according to the manufacturer’s instructions. Concentration and purity of the RNA samples were determined by Nanodrop ND 1000 spectrophotometer (Nanodrop Technologies, USA), and only the samples with A_260_/A_280_ ratio of 1.8–2.0 were used for cDNA synthesis. RNA integrity was further verified by 1.5% agarose gel electrophoresis. cDNA was synthesized from 1000 ng of the total RNA with the One Step PrimeScript miRNA cDNA Synthesis Kit (TaKaRa, Dalian, China).The cDNAs were diluted with ddH_2_O and used for qPCR.

### Selection of Candidate Reference Genes and Primer Design

Nine candidate genes, *eIF-4α*, *ACT7*, *TIP41*, *GAPDH*, *SAND*, *EF-1α*, *PP2A*, *TBP*, and *TUB*, which have been reported and used as good reference genes in qPCR analyses [8]–[12], [33], were selected for this study from TAIR database (http://www.arabidopsis.org). Potential homologues of the nine reference genes were identified from the transcriptome sequencing data of *O. javanica* (unpublished data) using BioEdit Sequence Alignment v 7.0.9 software [36]. Based on the sequences from the transcriptome sequencing data, nine candidate reference genes were cloned. PCR primers were designed using Primer 6.0 (Premier Biosoft International, Palo Alto, CA) and DNAMAN 6.0 (Lynnon Biosoft, USA) according to the manufacturer’s instructions. The specificity of the amplicon was verified by single band of expected size in a 1.5% agarose gel following electrophoresis and the presence of a single peak in the qPCR melting curve. The lists of primers used in qPCR and cloning are provided in Table 1 and Table S1.

### Quantitative RT-PCR Assay

Total RNA was extracted using RNAsimply total RNA Kit (Tiangen, Beijing, China) from leaves of the plant. cDNA was synthesized from 1000 ng of the total RNA using the PrimeScript RT reagent Kit (TaKaRa, Dalian, China). qPCR was performed in a 96 well plate on MyiQ Single color Real-Time PCR Detection System (Bio-rad, Hercules, USA) with SYBR Green I Mix (TaKaRa, Dalian, China). The reactions were performed in a final volume of 20 μL containing10 μLSYBR Green I Mix, 2 μL diluted cDNA, ddH_2_O, and a final primer concentration of 0.4 μM. The following amplification conditions were used: 95°C for 30 s, followed by 40 cycles at 95°C for 5 s, 60°C for 20 s. A dissociation curve from 65°C to 95°C was generated to verify primer specificity. The qPCR was designed according to MIQE guidelines [37]. Each assay included three technical and biological replicates, a no-template control, and a standard curve of six serial dilution points. Amplification efficiency (E) of the primer and correlation coefficient (R^2^) were calculated by standard curve method with five-fold dilution series for all the samples. The equation (% Efficiency = (10^[−1/slope]^−1)×100%) was used to calculate the E-value.

### Data Analysis

Expression levels of the nine reference genes in 24 samples (three biological duplicates and eight different conditions) were determined by the number of cycles (Cq) needed for the amplification-related fluorescence to reach a specific threshold level of detection. The raw data were listed in the Table S2. Following qPCR data collection, Cq values were converted to relative quantities using the formula: 2^−ΔCq^, in which ΔCq = the corresponding Cq value − minimum Cq. Relative quantities were used for the geNorm and the NormFinder, while BestKeeper analysis was performed based on raw Cq values [1], [3], [38], [39]. Expression stability of the reference genes was ranked using three different types of Microsoft Excel-based softwares: geNorm, NormFinder, and BestKeeper.

#### a) geNorm

The geNorm algorithm relies on the principle that the expression ratio of two ideal reference genes should be constant in a given sample set. Thus, whether a gene is constantly expressed or not can be balanced by the variation in expression ratios of two reference genes. The average pair-wise variation of a particular gene with all other reference genes can be defined as the expression stability value M. Reference gene with the lowest M value is considered as the most stable gene [3].

#### b) NormFinder

NormFinder program ranks all candidate reference genes based on intra- and inter-group variations and combines all the reference values into a stability value for each candidate reference gene [38].

#### c) BestKeeper

BestKeeper estimates gene expression stability for all individual reference genes based on the standard deviation (SD) and the coefficient of variation (CV) calculated with the Cq values of all candidate reference genes. Reference genes with the lowest SD and CV values are considered as the most stable genes [39].

## Results

### Assessment of Amplification Efficiency and Specificity

Based on the sequece of the genes cloned from *O. javanica*, the Specific primer pairs were designed for the candidate reference genes (*eIF-4α*, *ACT7, TIP41*, *GAPDH*, *SAND*, *EF-1α*, *PP2A*, *TBP*, and *TUB*), with the amplicon lengths ranging from 93 to 192 bp (Table 1). Melting curve analyses showed single peak for all the reactions. Expression was analyzed under various treatment conditions following qPCR. Amplification efficiencies were calculated from the standard curves with good linear relationships (R^2^>0.99); amplification efficiencies were 91.6% to 103.2% (Table 1).

### Cq Values of Candidate Reference Genes

**Figure 1:**
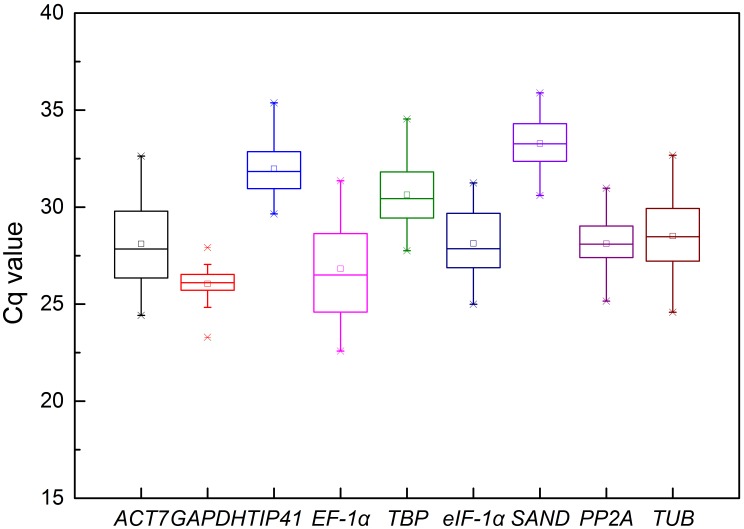
Cq values of candidate reference genes in all samples of *O. javanica*. The line across the box depicts median. The inside box depicts mean. The outside box is determined by the 25th and 75th percentiles. The whiskers are determined by the 5th and 95th percentiles. The asterisk represents outlier.

To provide an overview of the transcript levels of the nine candidate reference genes, expression levels were determined as quantification cycle (Cq) values (Table S2), and a distribution diagram was drawn (Fig. 1). Mean Cq values of the genes ranged from 26.05 (*GAPDH*)–33.28 (*SAND*), and the Cq values of all the tested samples were between 22 and 36. *TIP41, TBP* and *SAND* showed low expression levels with high Cq values, whereas, *eIF-4α*, *ACT7*, *GAPDH*, *EF-1α*, *PP2A*, and *TUB* showed moderate expression. The mean values of the candidate genes were close to their median values in majority of the genes, especially in *GAPDH*, *SAND*, *PP2A*, and *TUB*, indicating evenly distributed Cq values. The Cq values of *GAPDH* and *PP2A* distributed more centrally than other candidate genes (Fig. 1). Correspondingly, *GAPDH* and*PP2A* showed least variation in gene expression (standard deviation, SD = 0.78 and 1.27 for *GAPDH* and *PP2A*, respectively), while *EF-1α* (2.37) showed maximum variability across all the samples (Table S3).

### Determination of the Optimal Number of Reference Genes

**Figure 2:**
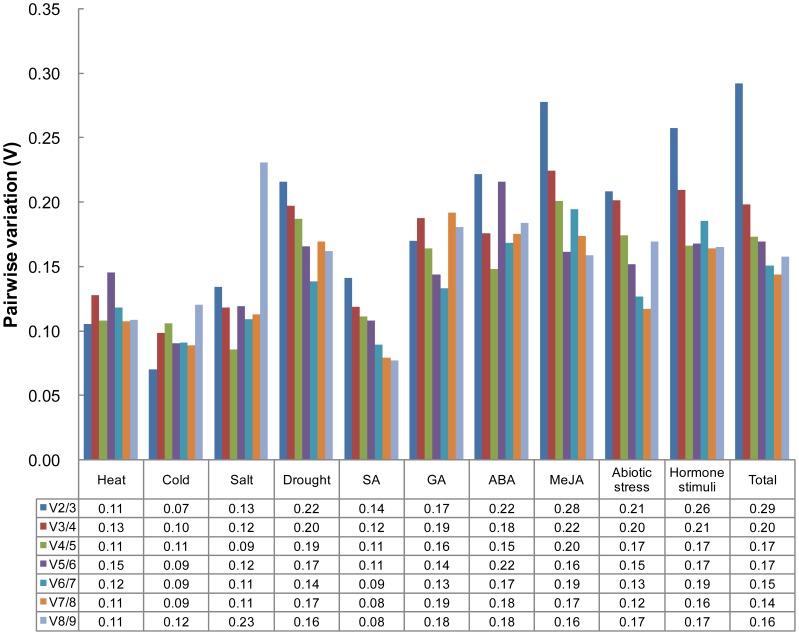
Determination of the optimal number of reference genes required for effective normalization. Pairwise variation (V_n/n +1_) analysis between the normalization factors (NF_n_ and NF_n +1_) was performed by the geNorm program to determine the optimal number of reference genes, and carried out for qPCR data normalization in various sample pools. GA: gibberellin; SA: salicylic acid; MeJA: methyl jasmonate; ABA: abscisic acid; Abiotic stress: heat, cold, salt, and drought; Hormone stimuli: SA, GA, ABA, and MeJA; Total: all sample.

geNorm calculates the pairwise variation (V_n/n+1_) between the sequential normalization factors (NF_n_ and NF_n+1_, n≥2) in a stepwise manner and determines the optimal number of reference genes required for accurate normalization. A large variation indicates the added reference gene has significant effect and is preferred to be included as a reliable normalization factor [3]. As shown in Fig. 2, inclusion of a third gene had no significant effect (V_2/3_ value was low) for heat, cold, salt, and SA treatments, hence two reference genes are sufficient for normalizing gene expression under such conditions. With a threshold of 0.15, four genes were needed for ABA treatment, five for GA and abiotic stress, and six for drought treatment.

### Expression Stability of Candidate Reference Genes

After simple comparison of the raw Cq values, we applied the three commonly used Microsoft Excel-based algorithms–geNorm, NormFinder, and BestKeeper–to further analyze stability of the nine candidate genes. In the program, eight different treatment sets were sorted into three groups: ‘Abiotic stress’ (heat, cold, salt, and drought), ‘Hormone stimuli’ (SA, GA, ABA, and MeJA), and ‘Total’ (samples in all treatments). Accordingly, eleven evaluation patterns were generated for both single stress and groups.

The ranks of the nine candidate reference genes based on their expression stability are shown in Table 2. In geNorm analysis, all the genes performed well under individual stress conditions with M values less than 1.5, which is the default limit. Under heat stress, all the nine candidate genes performed well in all the three softwares–geNorm, NormFinder, and BestKeeper. *EF-1α* and *ACT7* were found to be the two best reference genes among the nine candidate genes. Under cold stress, the nine candidate genes were confirmed to be stable in geNorm and BestKeeper. In Normfinder, *GAPDH* and *TUB* performed did not show satisfactory stability with values >0.04. *eIF-4α* and *ACT7* were the two most stable genes among the nine candidate genes under cold stress condition. Under salt stress, *TIP41*was the most stable by a comprehensive comparison. Under drought stress, *eIF-4α*, *TIP41*, and *ACT7* could be considered as the three best reference genes. In SA treatment, *TIP41* was the best reference gene even though all the other eight-candidate genes also expressed stably. In GA treatment, *eIF-4α* was best reference gene while *ACT7* and *EF-1α* were the least stable genes in geNorm and BestKeeper. In ABA treatment, *GAPDH* was the better reference gene while *ACT7*, *TUB*, and *EF-1α* proved to be the least stable genes by BestKeeper. In MeJA treatment, *SAND* was the most stable gene.

**Table 2:** Gene expression stability under individual stress ranked by geNorm, NormFinder and BestKeeper.

Treatments	Rank	geNorm		NormFinder		BestKeeper		
		Gene	Stability	Gene	Stability	Gene	SD	CV
Heat	1	*TBP*	0.31	*EF-1*	0.001	*GAPDH*	0.19	0.72
	2	*eIF-1α*	0.31	*TIP41*	0.001	*SAND*	0.68	1.96
	3	*EF-1α*	0.34	*ACT7*	0.001	*PP2A*	0.92	3.15
	4	*ACT7*	0.44	*eIF-4α*	0.002	*TUB*	0.95	3.13
	5	*TIP41*	0.50	*TBP*	0.002	*ACT7*	0.99	3.37
	6	*SAND*	0.64	*SAND*	0.003	*eIF-1α*	1.04	3.52
	7	*PP2A*	0.72	*GAPDH*	0.007	*EF-1α*	1.05	3.74
	8	*TUB*	0.78	*PP2A*	0.008	*TIP41*	1.08	3.22
	9	*GAPDH*	0.86	*TUB*	0.012	*TBP*	1.09	3.46
Cold	1	*ACT7*	0.16	*eIF-4α*	0.005	*GAPDH*	0.31	1.21
	2	*eIF-4α*	0.16	*ACT7*	0.006	*TIP41*	0.42	1.37
	3	*TBP*	0.20	*SAND*	0.007	*SAND*	0.62	1.96
	4	*SAND*	0.31	*EF-1α*	0.012	*TBP*	0.71	2.46
	5	*TUB*	0.41	*TIP41*	0.016	*eIF-4α*	0.76	2.92
	6	*EF-1α*	0.47	*PP2A*	0.016	*ACT7*	0.78	2.97
	7	*TIP41*	0.53	*TBP*	0.018	*PP2A*	0.79	2.89
	8	*PP2A*	0.59	*GAPDH*	0.044	*TUB*	1.12	4.11
	9	*GAPDH*	0.71	*TUB*	0.083	*EF-1α*	1.17	4.72
Salt	1	*TBP*	0.19	*TIP41*	0.002	*TIP41*	0.31	0.98
	2	*eIF-4α*	0.19	*TBP*	0.002	*eIF-4α*	0.66	2.38
	3	*PP2A*	0.34	*ACT7*	0.002	*SAND*	0.71	2.16
	4	*ACT7*	0.43	*EF-1α*	0.002	*TBP*	0.76	2.53
	5	*TUB*	0.46	*eIF-4α*	0.003	*PP2A*	0.81	2.97
	6	*SAND*	0.56	*SAND*	0.004	*TUB*	1.00	3.69
	7	*EF-1α*	0.64	*PP2A*	0.013	*ACT7*	1.06	3.82
	8	*TIP41*	0.72	*TUB*	0.113	*GAPDH*	1.15	4.56
	9	*GAPDH*	1.03	*GAPDH*	0.165	*EF-1α*	1.37	5.25
Drought	1	*ACT7*	0.60	*EF-1α*	0.001	*PP2A*	0.30	1.04
	2	*eIF-4α*	0.60	*eIF-4α*	0.001	*GAPDH*	0.37	1.41
	3	*TIP41*	0.67	*TIP41*	0.002	*SAND*	0.63	1.85
	4	*TUB*	0.77	*ACT7*	0.005	*TIP41*	0.92	2.82
	5	*GAPDH*	0.88	*TBP*	0.007	*TUB*	1.04	3.58
	6	*PP2A*	0.95	*GAPDH*	0.007	*ACT7*	1.11	3.92
	7	*SAND*	1.00	*PP2A*	0.011	*eIF-4α*	1.36	4.66
	8	*EF-1α*	1.11	*SAND*	0.013	*EF-1α*	1.60	5.81
	9	*TBP*	1.22	*TUB*	0.029	*TBP*	1.61	5.03
SA	1	*TIP41*	0.31	*EF-1α*	0.004	*PP2A*	0.28	1.02
	2	*eIF-4α*	0.31	*TBP*	0.006	*GAPDH*	0.32	1.24
	3	*PP2A*	0.40	*TIP41*	0.006	*TUB*	0.42	1.50
	4	*TBP*	0.46	*eIF-4α*	0.007	*SAND*	0.45	1.37
	5	*ACT7*	0.53	*SAND*	0.010	*TIP41*	0.58	1.84
	6	*GAPDH*	0.59	*PP2A*	0.011	*TBP*	0.59	1.98
	7	*EF-1α*	0.63	*GAPDH*	0.012	*EF-1α*	0.63	2.42
	8	*TUB*	0.66	*ACT7*	0.015	*eIF-4α*	0.64	2.34
	9	*SAND*	0.69	*TUB*	0.022	*ACT7*	0.75	2.75
GA	1	*eIF-4α*	0.47	*EF-1α*	0.006	*GAPDH*	0.37	1.41
	2	*SAND*	0.47	*GAPDH*	0.009	*SAND*	0.93	2.81
	3	*PP2A*	0.53	*eIF-4α*	0.011	*PP2A*	0.95	3.36
	4	*GAPDH*	0.67	*ACT7*	0.011	*TIP41*	0.96	3.04
	5	*TIP41*	0.77	*TIP41*	0.011	*eIF-4α*	1.07	3.80
	6	*TUB*	0.83	*PP2A*	0.012	*TUB*	1.42	5.06
	7	*TBP*	0.89	*TBP*	0.014	*TBP*	1.51	5.00
	8	*ACT7*	1.07	*SAND*	0.016	*ACT7*	2.31	8.30
	9	*EF-1α*	1.21	*TUB*	0.080	*EF-1α*	2.54	9.68
ABA	1	*TBP*	0.70	*EF-1α*	0.005	*GAPDH*	0.99	3.77
	2	*eIF-4α*	0.70	*PP2A*	0.005	*TIP41*	1.27	3.94
	3	*GAPDH*	0.73	*GAPDH*	0.006	*eIF-4α*	1.36	4.90
	4	*PP2A*	0.78	*ACT7*	0.023	*SAND*	1.40	4.25
	5	*SAND*	0.81	*SAND*	0.026	*TBP*	1.43	4.67
	6	*ACT7*	1.00	*eIF-4α*	0.033	*PP2A*	1.72	6.11
	7	*EF-1α*	1.08	*TBP*	0.057	*ACT7*	2.45	8.68
	8	*TIP41*	1.18	*TIP41*	0.065	*EF-1α*	2.57	9.61
	9	*TUB*	1.31	*TUB*	0.194	*TUB*	2.62	8.98
MeJA	1	*TIP41*	0.70	*EF-1α*	0.002	*GAPDH*	0.20	0.78
	2	*eIF-4α*	0.70	*ACT7*	0.003	*TIP41*	0.33	1.02
	3	*SAND*	0.83	*SAND*	0.003	*eIF-4α*	0.80	2.73
	4	*GAPDH*	0.91	*PP2A*	0.004	*SAND*	0.87	2.52
	5	*TBP*	0.99	*TIP41*	0.005	*TBP*	0.91	2.82
	6	*PP2A*	1.05	*eIF-4α*	0.005	*PP2A*	0.96	3.34
	7	*TUB*	1.18	*TBP*	0.006	*TUB*	1.44	4.91
	8	*ACT7*	1.28	*GAPDH*	0.022	*ACT7*	1.69	5.64
	9	*EF-1α*	1.35	*TUB*	0.050	*EF-1α*	1.99	6.75

Plants were submitted to the following treatments: Heat, Cold, Salt, Drought, Salicylic acid (SA), Gibberellins (GA), Methyl jasmonate (JA), Abscisic acid (ABA).

For complexity of the groups, it was more difficult to recognize the best reference gene (Table 3). In ‘Abiotic stress’ group, *ACT7*, *EF-1α*, and *eIF-4α* performed well in geNorm and NormFinder; however, *EF-1α* and *eIF-4α* showed a great variation in BestKeeper. Finally, we selected *ACT7* and *PP2A* as the two best reference genes. *ACT7* had been implied by the individual analysis of heat, cold, salt and drought treatments. In ‘Hormone stimuli’ group, *SAND* and *PP2A* performed well in all three softwares. In ‘Total’ group (data from all 24 samples), *PP2A* and *SAND* were the two most stable reference genes by a comprehensive view, although they were not the best by each algorithm.

**Table 3:** Gene expression stability under multiple stress ranked by geNorm, NormFinder and BestKeeper.

Group	Rank	geNorm		NormFinder		BestKeeper		
		Gene	Stability	Gene	Stability	Gene	SD	CV
Abiotic stress	1	*ACT7*	0.62	*PP2A*	0.008	*GAPDH*	0.64	2.44
	2	*EF-1α*	0.62	*EF-1α*	0.010	*PP2A*	0.93	3.31
	3	*eIF-4α*	0.67	*ACT7*	0.011	*SAND*	1.14	3.42
	4	*TIP41*	0.77	*eIF-4α*	0.012	*TIP41*	1.22	3.82
	5	*SAND*	0.85	*TIP41*	0.012	*ACT7*	1.37	4.93
	6	*TUB*	0.92	*SAND*	0.012	*TBP*	1.48	4.85
	7	*PP2A*	0.96	*TBP*	0.018	*TUB*	1.49	5.24
	8	*TBP*	1.00	*GAPDH*	0.034	*eIF-4α*	1.51	5.36
	9	*GAPDH*	1.14	*TUB*	0.045	*EF-1α*	1.70	6.38
Hormone stimuli	1	*SAND*	0.86	*EF-1α*	0.005	*GAPDH*	0.49	1.88
	2	*PP2A*	0.86	*PP2A*	0.006	*TIP41*	0.85	2.66
	3	*eIF-4α*	0.88	*SAND*	0.008	*SAND*	1.04	3.12
	4	*TBP*	0.92	*eIF-4α*	0.008	*PP2A*	1.08	3.83
	5	*TIP41*	0.95	*ACT7*	0.010	*eIF-4α*	1.20	4.28
	6	*GAPDH*	1.02	*GAPDH*	0.010	*TBP*	1.38	4.49
	7	*TUB*	1.14	*TBP*	0.011	*TUB*	1.73	6.05
	8	*ACT7*	1.23	*TIP41*	0.012	*ACT7*	2.04	7.22
	9	*EF-1α*	1.32	*TUB*	0.032	*EF-1α*	2.31	8.54
Total	1	*TIP41*	0.76	*EF-1α*	0.007	*GAPDH*	0.56	2.15
	2	*eIF-4α*	0.76	*PP2A*	0.008	*PP2A*	1.00	3.55
	3	*SAND*	0.89	*SAND*	0.011	*TIP41*	1.05	3.27
	4	*PP2A*	0.92	*ACT7*	0.011	*SAND*	1.09	3.28
	5	*TBP*	0.95	*eIF-4α*	0.012	*eIF-4α*	1.36	4.82
	6	*ACT7*	1.03	*TIP41*	0.013	*TBP*	1.44	4.70
	7	*TUB*	1.09	*TBP*	0.016	*TUB*	1.60	5.61
	8	*EF-1α*	1.15	*GAPDH*	0.022	*ACT7*	1.69	6.03
	9	*GAPDH*	1.24	*TUB*	0.039	*EF-1α*	2.00	7.45

The references genes stability was held in three groups: Abiotic stress: heat, cold, salt, and drought; Hormone stimuli: Salicylic acid (SA), Gibberellins (GA), Methyl jasmonate (JA), and Abscisic acid (ABA); Total: all samples.

### Reference Gene Validation

**Figure 3:**
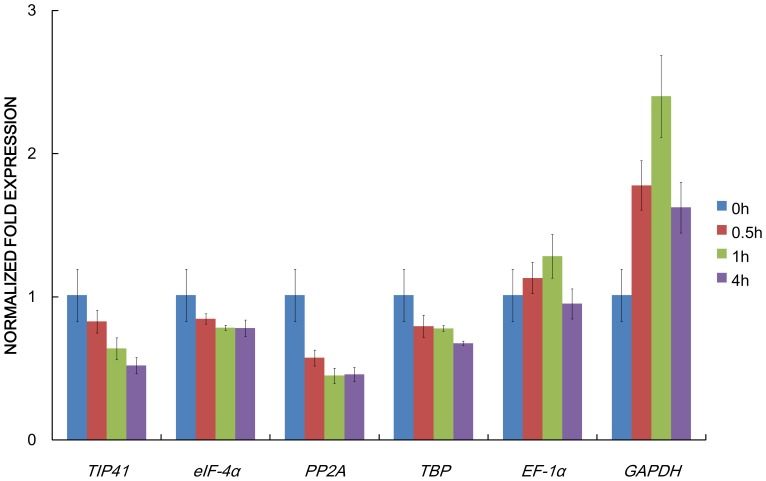
Relative quantification of *M6PR* gene expression using validated reference genes for normalization under salt treatment in *O. javanica*.

*M6PR* gene which was related to salt stress was selected to further evaluate the reliability of the reference genes by qPCR in *O. javanica* [40]. Relative expression of *M6PR* gene under salt treatment was calculated by using the genes of *TIP41*, *PP2A*, *eIF-4α*, *TBP*, *EF-1α* and *GAPDH* for normalization, respectively (Figure 3). When the most stable reference gene *TIP41* was used for normalization, the expression levels of *M6PR* decreased at 0.5, 1, and 4 h successively. Similar expression patterns were generated when the less stable reference genes *PP2A*, *eIF-4α*, and *TBP* were employed. When the least stable genes *EF-1α* and *GAPDH* were used for normalization, the expression patterns and transcript levels were very different. The expression levels increased and peaked at 1 h, then, decreased.

## Discussion

Gene expression analysis plays a significant role in biological research. Understanding the gene expression pattern not only provides insights into the complex regulatory networks but also identifies novel genes relevant to key biological processes [3]. Stability of reference gene expression is an elementary prerequisite for internal normalization of the target gene expression. Stable expression of the reference gene can exhibit either up- or down-regulation under some experimental conditions. Gene transcripts with invariant abundance under various environmental stimuli are essential reference points for accurate data analyses [11]. Previous researchers have showed that expression of a single reference gene may be constant in a given cell type or experimental condition, but could vary considerably in response to different stimuli. For the increasing qPCR application methods, the requirement for suitable reference genes has become increasingly demanding. *O. javanica* is a vegetable with some medicinal properties. It is worthy of doing further research on molecular level. In the current study, we performed a systematic evaluation of nine commonly used reference genes under different abiotic stress and hormone stimuli conditions in *O. javanica*, and ranked the genes according to their stability values calculated by using three commonly used algorithms.

A total of nine genes (*eIF-4α*, *ACT7*, *TIP41*, *GAPDH*, *SAND*, *EF-1α*, *PP2A*, *TBP*, and *TUB*) were selected as candidate reference genes for stable expression assessment tests. We cloned the nine candidate genes from *O. javanica*, then, primer pairs of qPCR were designed for the nine candidate reference genes. Single peak in the melting curve analyses confirmed the specificity of the primer pair. Expression data under different hormone stimuli (gibberellins, salicylic acid, methyl jasmonate, and abscisic acid), abiotic stresses (heat, cold, salt, and drought), and efficacy dilutions (1, 5, 5^2^, 5^3^, 5^4^×dilution) were collected following qPCR amplification and detection. Subsequently, corresponding standard curves were generated. The curves showed good linear relationships (R^2^>0.99) and their amplification efficiencies ranged from 91.6% to 103.2%. The results confirmed that the primer pairs and amplification conditions were appropriate for qPCR-based quantification.

Expression levels were determined as quantification cycle (Cq) values by qPCR. In the distribution diagram of the Cq values, we provided an overview of abundance of the nine candidate reference genes. The mean Cq values of the genes ranged from 26.05 (*GAPDH*)–33.28 (*SAND*), and the Cq values for all the tested samples were between 22 and 36. In this study, we used 40 cycles of amplification for qPCR; therefore, Cq values from 18 to 30 were considered as appropriate and reliable. Except *TIP41*, *TBP* and *SAND*, most of them were within the acceptable range. *TIP41*, *TBP*, and *SAND* showed the lowest expression and the highest Cq values; *eIF-4α*, *ACT7*, *GAPDH*, *EF-1α*, *PP2A*, and *TUB* were moderately expressed. Mean values of the candidate genes were closed to their median values for majority of the genes, especially for *GAPDH*, *SAND*, *PP2A*, and *TUB*, indicating that Cq values are evenly distributed. The Cq values of *GAPDH* and *PP2A* distributed more centrally than other candidate genes. The range of Cq values under different treatments indicated a considerable variability among the nine candidate reference genes. The least variation in gene expression across all the samples was observed in *GAPDH* and *PP2A* (<6 cycles; SD, of 0.78 and 1.27), while *EF-1α* showed the maximum variation (>8 cycles; SD = 2.37). The raw Cq value comparison is simple and can provide a rough estimate on stability of gene expression, but is not sufficient for accurate evaluation of expression pattern of the reference genes. Thus, three more sophisticated statistical analyses were used.

The results generated by geNorm and NormFinder were closer compared with the result generated by BestKeeper, especially for *GAPDH*. While geNorm and NormFinder correct for inter-sample variation, BestKeeper do not account for differences in RNA quality/input or reverse transcription (RT) efficiency across the samples, which can inflate the unexplained variance. In comparison to the pairwise approach used by the geNorm, the NormFinder approach where the top rank candidates were selected with minimal variation rather than correlated expression is least influenced by the co-regulated genes. Moreover, NormFinder takes systematic differences between the sample subgroups into consideration [3], [38], [39]. Comparison of the three algorithms could provide the most stable reference genes under specific experimental conditions.

In geNorm analysis, all genes performed well in both individual and multiple stress analysis with M values less than 1.5. By comparison with the three sort of rank, we obtained the most stable reference genes under specific experimental conditions. *ACT7* was chosen as the stable reference gene under heat, cold, and drought stress. *eIF-4α* could be marked as reference gene under cold, drought, and GA treatments. *TIP41* could be chosen for salt, drought and SA treatments. *EF-1α*, *GAPDH*, and *SAND* could be chosen as reference genes under heat, ABA, and MeJA treatments, respectively. *ACT7* and *PP2A* were picked as the most stable reference genes in ‘Abiotic stress’ group, while *PP2A* and *SAND* were selected for ‘Hormone stimuli’ and ‘Total’ group. We found that *PP2A* was the most stable gene across the entire sample groups; however, it was not the best under individual analysis. Previous studies have suggested that no single gene could express stably in all cell types and under all experimental conditions [41], [42]. Our present study further supports that normalization of target gene expression data with corresponding reference genes is essential to obtain accurate and reliable gene expression data profiles.

The transgenic plants over-expressed the mannitol-related genes exhibited more stress tolerance than wild type plants [40]. Many plants were transformed with a bacterial catabolic NAD-dependent mannitol-1P dehydrogenase which ordinarily converts mannitol-1P to fructose-6P. In an alternative approach, plants were transformed with the celery gene *M6PR* that usually converts mannose-6P to mannitol-1P as part of the path to mannitol biosynthesis [40], [43]. Here, under salt treatment, relative expression of *M6PR* was calculated by using *TIP41*, *PP2A*, *eIF-4α*, *TBP*, *EF-1α* and *GAPDH* for normalization, respectively. Expression levels of *M6PR* gene normalized by the most stable reference gene *TIP41* and less stable reference genes (*PP2A*, *eIF-4α*, and *TBP*) performed similar expression patterns. In contrast, when the least stable gene *EF-1α* and *GAPDH* was used for normalization, the expression patterns and transcript levels were very different. The results demonstrated that using an unstable reference gene generated biases could lead to misinterpretation of gene expression patterns.

## Supporting Information

Figure S1 **The phenotype of** ***Oenanthe javanica*** **.**
(PDF)

Table S1 **Primer sequences.**
(PDF)

Table S2 **Raw Cq values in** ***Oenanthe javanica.*** Plants were submitted to the following treatments: Heat, Cold, Salt, Drought, Salicylic acid (SA), Gibberellins (GA), Methyl jasmonate (JA), Abscisic acid (ABA); Control: samples without treatment.
(PDF)

Table S3 **Data statistics of Cq values of candidate reference genes.**
(PDF)
