# Supplementary figures and images for: Selection of Suitable Reference Genes for qPCR Normalization under Abiotic Stresses in Oenanthe javanica (BI.) DC
Source: PLoS One. 2014 Mar 20;9(3):e92262. doi: 10.1371/journal.pone.0092262 (PMC3961309; doi:10.1371/journal.pone.0092262)

**Figure S1** The phenotype of *Oenanthe javanica*.

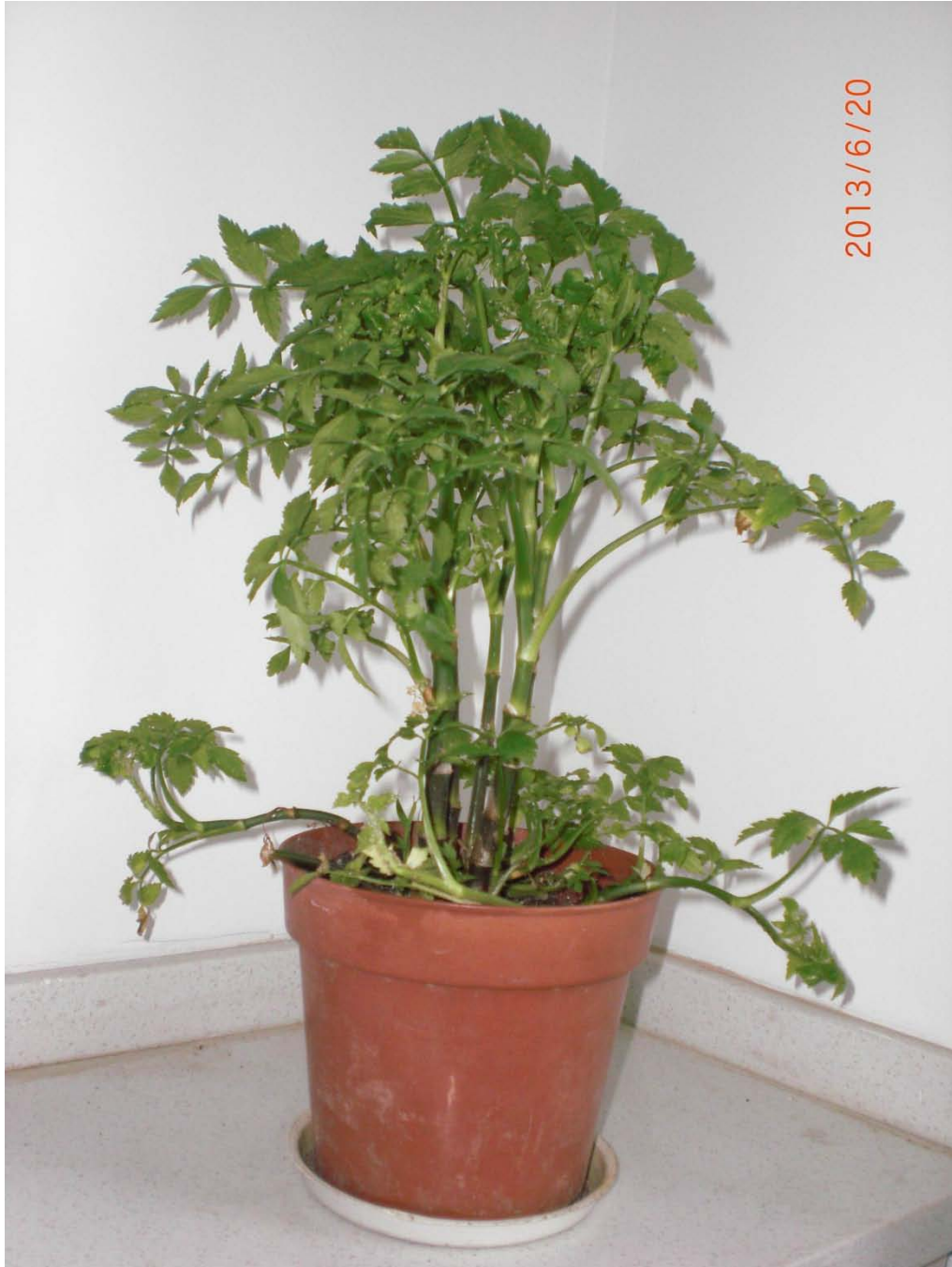

Supplement: Figure S1 — The phenotype of Oenanthe javanica . (PDF) [file pone.0092262.s001.pdf]
