# Supplementary material for: Selection of Suitable Reference Genes for qPCR Normalization under Abiotic Stresses in Oenanthe javanica (BI.) DC
Source: PLoS One. 2014 Mar 20;9(3):e92262. doi: 10.1371/journal.pone.0092262 (PMC3961309; doi:10.1371/journal.pone.0092262)
Supplement: Table S1 — Primer sequences. (PDF) [file pone.0092262.s002.pdf]

Table S1 Primer sequences

Gene symbol	Primer sequence (5'–3') forward/reverse	Amplicon length (bp)
eIF-4α	CAACTCCTGCGGCGTAGAT/ ACCAACATCCTGTCATCATCCT	1265
ACT7	GCGATAATGGAACTGGAATGGT/ TGCTGGAAGGTACTGAGAG	1033
TIP41	CGGTAATTAAGTCGTCGATGGA/ CGCAGCACAATCAGAAGGATA	800
GAPDH	ACTAACATCAGGCTTCCATCCA/ TCGTTATCGTACCAGGCTACAA	1330
SAND	ATATGCTCTTGCTTGCCAACT/ AGGTTAGCCTCAATGAAGACTT	942
EF-1α	TGAGCGTGAGCGTGGTAT/ CATAGGCTTGGTTGGAATCATC	1000
PP2A	AGTTGGATATGTGTTGCGTGTT/ GCTGAAGATGTTGGTTCCTCAA	1321
TBP	CCCATCTAATCTCTCCCATTCCT/ AATCTTAACACAGCCACGACAA	1058
TUB	AAGTATGTTGGCAAGTCGGATT/ TCCTCCTCGTCAGCAGTTG	1199
M6PR	ACACAGAGGCAGTATCACCAT/ AACTTGGCAGGTTGATTAGTCC	997
M6PR-qPCR	ACTAGAGATTGCTTGGCGTATGCT/ AGTGGTGTGTGGGCTGTAATGC	134
