# Supplementary material for: Selection of Suitable Reference Genes for qPCR Normalization under Abiotic Stresses in Oenanthe javanica (BI.) DC
Source: PLoS One. 2014 Mar 20;9(3):e92262. doi: 10.1371/journal.pone.0092262 (PMC3961309; doi:10.1371/journal.pone.0092262)
Supplement: Table S2 — Raw Cq values in Oenanthe javanica. Plants were submitted to the following treatments: Heat, Cold, Salt, Drought, Salicylic acid (SA), Gibberellins (GA), Methyl jasmonate (JA), Abscisic acid (ABA); Control: samples without treatment. (PDF) [file pone.0092262.s003.pdf]

**Table S2** Raw Cq values in *Oenanthe javanica*

Plants were submitted to the following treatments: Heat, Cold, Salt, Drought, Salicylic acid (SA), Gibberellins (GA), Methyl jasmonate (JA), Abscisic acid (ABA); Control: samples without treatment.

| | *ACT7* | *GAPDH* | *TIP41* | *EF-1α* | *TBP* | *eIF-4α* | *SAND* | *PP2A* | *TUB* |
|---|---|---|---|---|---|---|---|---|---|
| Control-1 | 21.92 | 21.85 | 25.72 | 19.78 | 23.10 | 21.92 | 27.16 | 22.69 | 23.92 |
| Control-2 | 22.13 | 22.13 | 25.66 | 20.01 | 23.21 | 22.06 | 27.31 | 22.80 | 24.04 |
| Control-3 | 22.09 | 22.04 | 25.83 | 20.02 | 23.18 | 22.06 | 27.19 | 22.81 | 24.04 |
| Heat-1 | 30.48 | 26.51 | 34.10 | 28.71 | 32.49 | 30.08 | 35.84 | 30.22 | 30.66 |
| Heat-2 | 30.75 | 26.72 | 35.38 | 29.09 | 31.77 | 30.28 | 35.00 | 30.98 | 31.91 |
| Heat-3 | 30.05 | 26.27 | 34.56 | 28.51 | 31.99 | 29.86 | 35.76 | 30.67 | 32.29 |
| Heat-4 | 28.26 | 26.47 | 31.93 | 26.38 | 30.04 | 28.35 | 33.25 | 27.58 | 29.17 |
| Heat-5 | 27.80 | 26.38 | 31.89 | 26.36 | 29.60 | 27.84 | 34.32 | 28.19 | 30.10 |
| Heat-6 | 27.78 | 26.35 | 31.70 | 26.53 | 29.81 | 28.08 | 33.72 | 28.85 | 28.27 |
| Heat-7 | 29.79 | 26.71 | 33.54 | 28.44 | 32.20 | 30.52 | 34.02 | 28.57 | 30.83 |
| Heat-8 | 30.20 | 26.65 | 33.89 | 28.60 | 32.04 | 30.65 | 34.80 | 29.04 | 29.76 |
| Heat-9 | 29.79 | 27.05 | 34.12 | 29.30 | 33.11 | 31.25 | 34.62 | 29.06 | 30.19 |
| Cold-1 | 25.35 | 25.68 | 29.66 | 23.60 | 27.76 | 25.07 | 30.83 | 26.02 | 25.94 |
| Cold-2 | 25.19 | 25.85 | 29.67 | 23.64 | 27.84 | 24.99 | 30.75 | 26.35 | 25.78 |
| Cold-3 | 25.48 | 25.64 | 29.77 | 23.65 | 27.80 | 25.16 | 30.60 | 25.79 | 25.95 |
| Cold-4 | 25.92 | 26.52 | 30.55 | 24.13 | 28.99 | 26.02 | 31.81 | 27.89 | 27.52 |
| Cold-5 | 26.07 | 25.86 | 30.65 | 23.91 | 28.69 | 25.93 | 31.56 | 27.72 | 26.84 |
| Cold-6 | 25.81 | 26.37 | 30.45 | 24.01 | 28.64 | 25.92 | 32.11 | 28.03 | 27.22 |
| Cold-7 | 27.30 | 25.46 | 30.63 | 26.37 | 29.68 | 27.24 | 32.55 | 27.10 | 28.94 |
| Cold-8 | 27.42 | 25.46 | 30.85 | 26.23 | 29.70 | 27.32 | 32.09 | 28.10 | 29.10 |
| Cold-9 | 27.46 | 25.35 | 30.68 | 26.74 | 29.95 | 27.13 | 32.38 | 27.76 | 28.94 |
| Salt-1 | 25.98 | 25.58 | 30.96 | 24.01 | 28.84 | 26.50 | 32.04 | 25.97 | 26.01 |
| Salt-2 | 26.16 | 25.68 | 30.76 | 24.08 | 28.61 | 26.58 | 32.43 | 26.09 | 25.75 |
| Salt-3 | 26.03 | 25.76 | 31.10 | 24.31 | 28.93 | 26.69 | 31.98 | 26.33 | 25.54 |
| Salt-4 | 28.00 | 26.50 | 31.86 | 25.95 | 30.06 | 27.71 | 32.74 | 26.97 | 26.60 |
| Salt-5 | 27.79 | 26.70 | 31.81 | 26.19 | 30.47 | 27.83 | 32.97 | 27.51 | 27.21 |
| Salt-6 | 28.15 | 26.69 | 31.54 | 26.41 | 30.14 | 27.88 | 32.82 | 27.49 | 27.03 |
| Salt-7 | 28.86 | 23.74 | 31.62 | 28.18 | 31.09 | 28.45 | 34.38 | 28.26 | 28.56 |
| Salt-8 | 29.12 | 23.64 | 31.26 | 28.23 | 30.70 | 28.30 | 34.69 | 27.73 | 28.53 |
| Salt-9 | 28.67 | 23.29 | 31.34 | 27.83 | 30.53 | 28.26 | 32.45 | 28.89 | 28.54 |
| Drought-1 | 26.75 | 25.95 | 31.18 | 25.11 | 29.70 | 27.08 | 33.64 | 28.33 | 27.22 |
| Drought-2 | 26.48 | 25.73 | 30.89 | 24.97 | 29.62 | 27.03 | 33.08 | 28.74 | 27.68 |
| Drought-3 | 26.73 | 25.95 | 31.41 | 25.24 | 30.00 | 27.27 | 33.54 | 28.18 | 27.45 |
| Drought-4 | 28.62 | 26.55 | 33.66 | 27.64 | 34.54 | 30.13 | 34.41 | 29.19 | 29.13 |

| | | | | | | | | | |
|---|---|---|---|---|---|---|---|---|---|
| Drought-5 | 28.42 | 26.78 | 33.60 | 27.62 | 34.26 | 29.90 | 34.14 | 28.33 | 28.62 |
| Drought-6 | 28.85 | 26.99 | 33.44 | 27.49 | 34.40 | 30.68 | 33.09 | 28.71 | 29.72 |
| Drought-7 | 29.42 | 26.14 | 32.60 | 29.80 | 31.71 | 29.97 | 32.62 | 28.40 | 30.84 |
| Drought-8 | 29.77 | 26.44 | 32.99 | 29.71 | 32.17 | 29.89 | 34.55 | 29.02 | 30.53 |
| Drought-9 | 29.83 | 26.72 | 33.10 | 29.92 | 31.87 | 30.52 | 34.74 | 28.35 | 28.98 |
| SA-1 | 27.82 | 26.30 | 31.50 | 26.49 | 30.00 | 27.33 | 32.30 | 27.52 | 29.10 |
| SA-2 | 27.66 | 26.30 | 31.46 | 26.69 | 29.65 | 26.88 | 32.96 | 27.30 | 27.79 |
| SA-3 | 27.46 | 26.00 | 30.96 | 26.27 | 29.78 | 26.80 | 32.34 | 27.34 | 27.73 |
| SA-4 | 27.93 | 26.09 | 31.97 | 26.06 | 31.39 | 28.38 | 32.89 | 27.76 | 28.02 |
| SA-5 | 27.87 | 25.76 | 32.62 | 25.88 | 30.44 | 28.45 | 32.78 | 28.37 | 27.74 |
| SA-6 | 28.10 | 26.67 | 32.79 | 26.65 | 30.43 | 28.33 | 33.72 | 28.05 | 27.96 |
| SA-7 | 26.06 | 25.56 | 30.90 | 24.52 | 29.03 | 26.86 | 32.04 | 27.56 | 27.22 |
| SA-8 | 26.23 | 25.55 | 31.25 | 24.65 | 29.29 | 26.81 | 32.89 | 27.59 | 27.16 |
| SA-9 | 26.08 | 26.52 | 30.85 | 25.62 | 29.08 | 26.98 | 33.93 | 27.25 | 28.42 |
| GA-1 | 25.41 | 25.45 | 30.22 | 23.83 | 27.97 | 26.65 | 31.85 | 27.12 | 25.77 |
| GA-2 | 25.48 | 26.01 | 30.26 | 24.19 | 28.30 | 26.89 | 32.43 | 27.68 | 26.40 |
| GA-3 | 25.60 | 25.97 | 30.34 | 24.25 | 28.72 | 26.75 | 32.08 | 26.84 | 26.41 |
| GA-4 | 31.82 | 26.80 | 32.63 | 29.87 | 32.47 | 29.67 | 34.29 | 30.45 | 30.48 |
| GA-5 | 31.15 | 26.93 | 32.61 | 30.39 | 31.64 | 29.87 | 35.10 | 29.25 | 29.63 |
| GA-6 | 30.85 | 26.49 | 32.34 | 29.98 | 33.21 | 29.35 | 33.57 | 29.05 | 30.27 |
| GA-7 | 26.65 | 26.08 | 33.00 | 24.52 | 29.60 | 27.67 | 32.55 | 27.66 | 27.79 |
| GA-8 | 26.66 | 25.95 | 32.01 | 24.51 | 29.89 | 27.64 | 32.44 | 27.34 | 27.60 |
| GA-9 | 26.69 | 26.36 | 32.03 | 24.86 | 30.52 | 27.80 | 32.06 | 28.09 | 27.69 |
| ABA-1 | 30.04 | 27.02 | 33.96 | 28.68 | 32.32 | 29.69 | 34.22 | 29.51 | 31.69 |
| ABA-2 | 30.08 | 26.85 | 33.93 | 28.39 | 31.19 | 29.58 | 33.64 | 30.18 | 29.71 |
| ABA-3 | 30.53 | 27.92 | 34.49 | 29.57 | 33.47 | 29.75 | 34.08 | 30.07 | 31.45 |
| ABA-4 | 24.42 | 24.83 | 31.11 | 22.89 | 27.93 | 25.70 | 31.10 | 25.54 | 25.49 |
| ABA-5 | 24.64 | 24.34 | 31.47 | 22.58 | 28.69 | 25.73 | 30.67 | 25.15 | 24.58 |
| ABA-6 | 24.55 | 24.90 | 30.89 | 23.12 | 28.64 | 25.79 | 31.06 | 25.93 | 25.77 |
| ABA-7 | 30.42 | 26.59 | 31.26 | 28.67 | 30.75 | 27.66 | 34.04 | 29.09 | 29.75 |
| ABA-8 | 29.46 | 26.65 | 31.29 | 28.23 | 31.19 | 27.41 | 34.69 | 28.79 | 32.68 |
| ABA-9 | 29.73 | 26.43 | 31.62 | 28.29 | 30.82 | 27.48 | 33.92 | 28.80 | 31.79 |
| MeJA-1 | 31.48 | 25.90 | 32.84 | 30.83 | 31.76 | 29.09 | 34.85 | 30.47 | 30.28 |
| MeJA-2 | 29.80 | 25.82 | 32.88 | 31.11 | 33.28 | 29.12 | 34.33 | 29.71 | 31.10 |
| MeJA-3 | 31.05 | 25.96 | 32.45 | 30.52 | 31.64 | 28.99 | 35.20 | 29.51 | 31.57 |
| MeJA-4 | 27.39 | 26.40 | 32.15 | 26.54 | 31.62 | 28.39 | 33.28 | 27.47 | 27.28 |
| MeJA-5 | 27.69 | 26.13 | 31.97 | 26.31 | 30.45 | 28.78 | 33.68 | 27.09 | 26.74 |
| MeJA-6 | 27.61 | 26.00 | 32.40 | 26.59 | 31.61 | 28.76 | 33.56 | 27.77 | 27.39 |
| MeJA-7 | 31.02 | 25.42 | 32.77 | 31.01 | 34.27 | 30.81 | 33.42 | 29.28 | 30.27 |
| MeJA-8 | 31.57 | 25.74 | 33.20 | 31.36 | 33.10 | 30.90 | 35.89 | 28.42 | 29.33 |
| MeJA-9 | 32.63 | 25.70 | 32.87 | 30.88 | 32.36 | 30.28 | 35.69 | 29.19 | 29.69 |
