# Supplementary material for: Selection of Suitable Reference Genes for qPCR Normalization under Abiotic Stresses in Oenanthe javanica (BI.) DC
Source: PLoS One. 2014 Mar 20;9(3):e92262. doi: 10.1371/journal.pone.0092262 (PMC3961309; doi:10.1371/journal.pone.0092262)
Supplement: Table S3 — Data statistics of Cq values of candidate reference genes. (PDF) [file pone.0092262.s004.pdf]

Table S3 Data statistics of Cq values of candidate reference genes

	**N total**	**Median**	**Mean**	**Standard Deviation**	**Minimum**	**Maximum**
ACT7	72	27.85	28.11	2.02	24.42	32.63
GAPDH	72	26.11	26.05	0.78	23.29	27.92
TIP41	72	31.84	31.98	1.28	29.66	35.38
EF-1α	72	26.51	26.83	2.37	22.58	31.36
TBP	72	30.44	30.64	1.74	27.76	34.54
eIF-4α	72	27.86	28.13	1.61	24.99	31.25
SAND	72	33.27	33.28	1.31	30.60	35.89
PP2A	72	28.10	28.12	1.27	25.15	30.98
TUB	72	28.47	28.52	1.91	24.58	32.68
